# Aqueous Dispersion of Xenes by Liquid Phase Exfoliation of Monoelemental Crystals in Melamine Solution

**DOI:** 10.1002/chem.202403770

**Published:** 2024-12-11

**Authors:** Alexia Vaso, Eleni Tegkelidi, Angela S. Kaloudi, Dimitrios P. Gournis, Vasileios Tzitzios, Nikos Boukos, Argiris Kolokithas Ntoukas, Vasilios I. Georgakilas

**Affiliations:** ^1^ Department of Materials Science University of Patras Rio 26504 Greece; ^2^ Department of Materials Science and Engineering University of Ioannina Ioannina GR-45110 Greece; ^3^ School of Chemical and Environmental Engineering Technical University of Crete Chania GR-73100 Greece; ^4^ Institute of GeoEnergy Foundation for Research and Technology-Hellas Chania GR-73100 Greece; ^5^ Institute of Nanoscience and Nanotechnology NCSR “Demokritos” Agia Paraskevi 15341 Greece; ^6^ Regional Centre of Advanced Technologies and Materials Czech Advanced Technology and Research Institute (CATRIN) Palacký University Olomouc Czechia; ^7^ Department of Pharmacy School of Health Sciences University of Patras Patras 26504 Greece

**Keywords:** 2D nanostructures, Liquid exfoliation, Bismuthene, Silicene, Stanene, Tellurene, Aqueous dispersion, Xenes

## Abstract

Αfter the impressive evolution of graphene and its derivatives, a large number of two dimensional (2D) materials with important optical and electrical properties have been successfully fabricated. Liquid phase exfoliation (LPE) of layered and non‐layered materials has become a widely applied method for the preparation of 2D nanostructures with an extensive variety of applications. However, in most cases organic solvents are used as liquid phase which are often toxic and environmentally unfriendly and lead to low yields. In this work, we present water as a suitable liquid phase and dispersion medium for the exfoliation of layered and non‐layered monoelemental solids from IV_A_, V_A_ and VI_A_ groups of the periodic table, such as silicon, tin, bismuth and tellurium. The 2D nanostructures, silicene, stanene, bismuthene and tellurene are therefore prepared by a completely sustainable and environmentally friendly method. The prepared Xenes, as they are called, are fully characterized by microscopic and spectroscopic techniques.

## Graphical abstract

Bismouth, silicon, tellurium and tin powders are effectively exfoliated in water solution of melamine affording 2D nanostructures. These characteristic 2D Xenes are dispersed in water forming stable colloidal solutions.

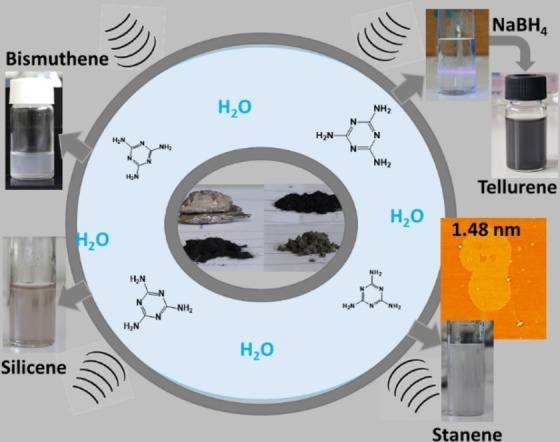

## 1 Introduction

The revolutionary development of nanomaterials has introduced many new top‐down processes aimed at controlling the size and shape of materials or chemical functionalization. Ultrasonic assisted liquid phase exfoliation (LPE), mechanochemical or microwave treatment and shear mixing, have been used on a wide variety of solids.^[1]^ Low power bath or high‐power horn sonication has evolved into a useful, widely applied process for reducing the size of materials from micro to nanometers. To date, ultrasonication has become a powerful tool for the production of graphene from graphite^[2]^ and a series of 2D nanostructures from van der Waals solids^[3]^ such as MoS_2_, h‐BN, monoelemental solids such as black phosphorous,^[4]^ bismuth,^[5]^ antimony,^[6]^ tellurium,^[7]^ or non van der Waals such as hematite,[^8^, ^9^] magnetite,^[9]^ ilmenite,^[10]^ silicon,^[11]^ tin,^[12]^ sulfides, (FeS_2_), oxides (WO_3_), and carbides (TiC, WC, SiC).^[3]^ Dimethyl formamide (DMF), N‐methyl pyrrolidone (NMP) and other specific organic solvents were mainly used, which are often associated with low yields and the disadvantages of toxicity, high boiling point and environmentally unfavorable use.^[3]^ Οn the other hand, a major effort is being made to introduce green solvents that will make a decisive contribution to the sustainability of 2D materials.^[13]^ Cyrene was the first organic green solvent introduced by Salavagione et al with very good performance for the production of high‐quality graphene. It is a non‐toxic and environmentally friendly derivative of cellulose‐rich biomass.^[14]^

Cyrene then proved to be an effective solvent for the exfoliation of transition metal dichalcogenides (TMD) such as molybdenum and tungsten sulfides (MoS_2_, WS_2_), yielding very good quality nanosheets with good performance. The main disadvantage of cyrene, however, is its high boiling point which makes it difficult to remove it from the final products.^[15]^ Recently, the need for sustainable 2D materials has also given rise to new green solvents such as methyl‐5‐(dimethylamino)‐2‐methyl‐5‐oxopentanoate, commonly named as polarclean, dimethyl 2‐methylglutarate (Iris) for the exfoliation of MoS_2_, WS_2_, graphite and germanium selenide (GeSe)[^16^, ^17^, ^18^] or plant extract for MoS_2_.^[19]^ 2D materials are of great importance for a variety of applications involving catalytic processes and physicochemical properties such as adsorption and chemisorption, thanks to their extremely large active surface area per unit mass and their mechanical stability in contrast to large surface area nanoparticles.[^20^, ^21^, ^22^, ^23^]

Our group, in an effort to develop sustainable, and environmentally friendly, low‐cost methods for the formation of nanostructures, introduced the use of natural aqueous extracts like curcuma, cinnamon or red pepper to produce graphene nanosheets from graphite^[24]^ and recently the use of aqueous melamine solution to exfoliate non van der Waals solids such as hematite and magnetite ore, with high yields.^[9]^ In this work, we describe the sonication‐assisted LPE of selected monoelemental materials from group IV_A_, V_A_ and VI_A_, in melamine aqueous solution and the formation of stable aqueous dispersion of 2D nanostructures, the as called Xenes.

Xenes have excellent biocompatibility and thanks to their photonic, physical, and chemical properties, they have been considered ideal candidates for diagnosis and therapeutic systems in bioapplications.^[25]^ The naturally derived, van der Waals solids, bismuth (Bi) and tellurium (Te) and the non van der Waals, silicon (Si) and tin (Sn) are discussed here for the formation of 2D nanostructures with interesting optoelectronic properties.

Bulk Bi has a rhombohedral structure consisting of six‐membered rings of Bi atoms. Monolayer and few layers bismuthene nanosheets have been prepared by LPE in IPA,^[26]^ NMP,[^27^, ^28^] ball milling for thermoelectric applications.^[29]^ Recently, bismuthene was prepared by the reduction of bismuth chloride from sodium borohydride (NaBH_4_) in 2‐ethoxyethanol and was successfully tested as a catalyst in the electroreduction of CO_2_.^[30]^ NaBH_4_ has also been shown to assist the exfoliation of Bi powder in nanosheets which were further used in bioapplications.^[31]^ Te has a chain‐like structure that is characterized by weak forces between the chains and stronger Te−Te forces in the chains.^[32]^ 2D few layer tellurene nanosheets have been formed by simple mechanical stirring in NMP,^[33]^ or sonication assisted LPE in IPA.^[34]^ Τhe very interesting behavior of tellurene as a topological insulator has been recently experimentally demonstrated. Tellurene has very intrusive properties such as variable band gap, piezoelectricity, photoresponse, leading to a number of inventions such as photodetectors, devices for energy applications.[^35^, ^36^] Bulk Si is one of the most used materials in electronic and optoelectronic technology. Its crystal has a diamond‐like structure, where the atoms are covalently bonded together. Si at the nanoscale has been studied for many applications[^37^, ^38^] such as in photovoltaics, sensors, batteries,^[39]^ electronic devices, photocatalytic hydrogen production.^[40]^ Low‐dimensional Si has been prepared by few methods, with low yields and often high costs, including chemical vapor deposition, chemical etching, such as the treatment of CaSi_2_ with HCl.[^11^, ^41^, ^42^] Recently, Wang et al demonstrated the exfoliation of Si powder on nanosheets by a cryo‐mediated liquid phase exfoliation in ethanol‐water mixture.^[40]^ Tin occurs mainly in two allotropic forms. β‐Tin has a body‐centered tetragonal crystal structure and is metallic in character and the a‐form, gray tin, which is stable below 13.2 °C, has a cubic diamond crystal structure and is semiconductor.^[43]^ The 2D analogue that is called stanene, to date has been formed by the epitaxial growth technique.^[44]^ Stanene has attracted the interest of scientists due to its topological insulator property. This class of materials exhibits electrical conductivity only at their outer edges or surfaces. When they are one atom thick, their edges exhibit superconductivity, forcing electrons to move in defined lanes without resistance.^[45]^

The formulation and dispersion of nanomaterials in aqueous solutions will give a significant boost to the development of new sustainable functional inks. Water‐based functional inks are composite materials that allow the coating of specific substrates with semiconducting, dielectric, magnetic nanomaterials in the form of thin films, circuits, or detailed devices. These inks can contribute significantly to the development of applications such as flexible electronics, sensors, coatings, energy storage systems, etc.

## 2 Results and Discussion

After the successful exfoliation of graphite,^[24]^ hematite and magnetite^[9]^ in aqueous solution of organic compounds and the high yield formation of graphene, hematene and magnetene nanoplatelets, four characteristic solids were selected as precursors for the preparation of the analogous 2D nanostructures; metalloid and semiconducting Si and metallic Sn from group IV_A_, post transition metal and pnictogen Bi from group V_A_, and metalloid Te from group VI_A_.

**Figure 1:**
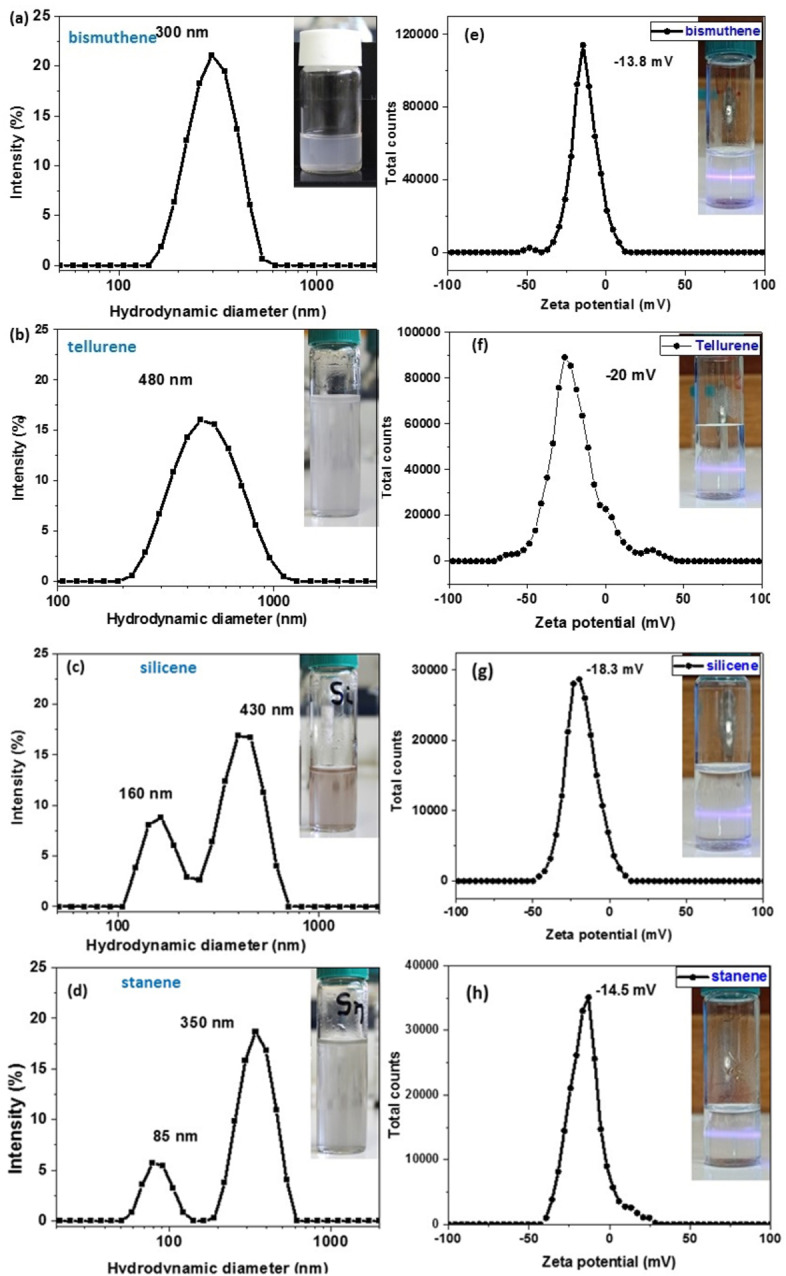
DLS measurements of a) bismuthene b) tellurene c) silicene d) stanene nanoplatelets (in the insert photos the dispersions of Xenes in water). Zeta potential measurements of e) bismuthene f) tellurene g) silicene h) stanene nanoplatelets (in the insert photos the dispersion of Xenes in water showing the Tyndall effect).

The exfoliation of the solids was achieved after 2 hours of sonication of the powders in a solution of melamine in water. The exfoliated materials were filtered and washed with water to remove the excess melamine. The nanostructures were redispersed in pure water as shown in Figure 1, in two different concentrations. In the diluted samples, the colloidal dispersion of the nanostructures is observed with the Tyndal effect. Dynamic light scattering (DLS) measurements provided an estimate of the mean size of the nanostructures formed, which ranges from 300 to 500 nm (Figure 1a–d). The *ζ* potential of the nanosheets range between 13.8 and 20 mV, while their charge was negative (Figure 1e–h). The low values of ζ potential indicate that the stability of the nanosheets in water is due more to steric repulsions that created from the adsorbed melamine molecules and less to electrostatic interactions.

Following the suggestion of Coleman et al,^[3]^ the 2D products of the van der Waals solids (bismuthene and tellurene) are then called nanosheets and that of the non van der Waals (silicene and stanene) are then called nanoplatelets. The XRD patterns of the 2D nanostructures consist of peaks corresponding to major planes like those of the 3D crystals (see Figure 5 and Figures S1, S3, S5), slightly shifted. Although the sonication treatment was carried out with deoxygenated water, XRD pattern indicated that the tellurene nanosheets were partially oxidized during exfoliation.

**Figure 2:**
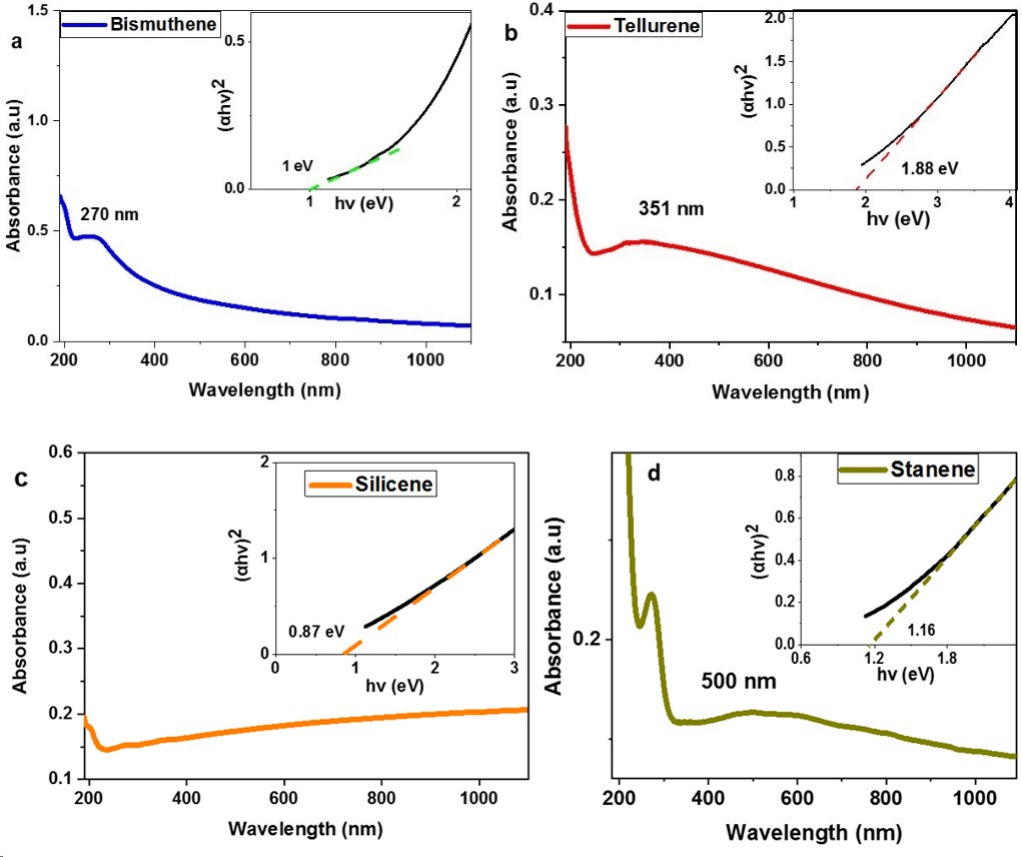
UV‐Vis spectra of aqueous dispersion of a) Bi and b) Te nanosheets, c) Si and d) Sn nanoplatelets. The inset images represent the Tauc plot and optical band gap estimation of the 2D products.

The colloidal dispersion of the 2D nanostructures in water greatly facilitates their characterization by UV‐Vis spectroscopy. The aqueous dispersion of bismuthene nanosheets showed strong absorption in the UV region with λ_max_ at 270 nm, which decreases significantly in the visible region. The optical band gap corresponding to this material was estimated by Tauc plot at 1.0 eV (Figure 2a). Ιn the literature, bismuthene has been reported as a semiconductor with a theoretical band gap between 0.36 and 0.99 eV and experimentally estimated to 0.61 and 0.8 eV elsewhere.[^46^, ^47^] The absorption spectrum of tellurene nanosheets in water dispersion showed a broad absorption covering the whole visible region and having λ_max_ at 351 nm and an optical band gap at 1.88 eV (Figure 2b). Zhang et al prepared tellurene nanosheets in IPA and the UV‐Vis spectrum showed a similar broad band but with lower absorbance above 800 nm and calculated the energy band gap at 1.88 eV.^[34]^ Silicene nanoplatelets in aqueous dispersion showed a broad absorption covering the visible region and extending into the infrared and estimated optical band gap at 0.87 eV (Figure 2c).^[11]^ Finally, stanene nanoplatelets appears to absorb in the UV region, while this absorption extents into the visible region but with very low absorptivity. The optical band gap corresponding to stanene was estimated at 1.16 eV (Figure 2d).

**Figure 3:**
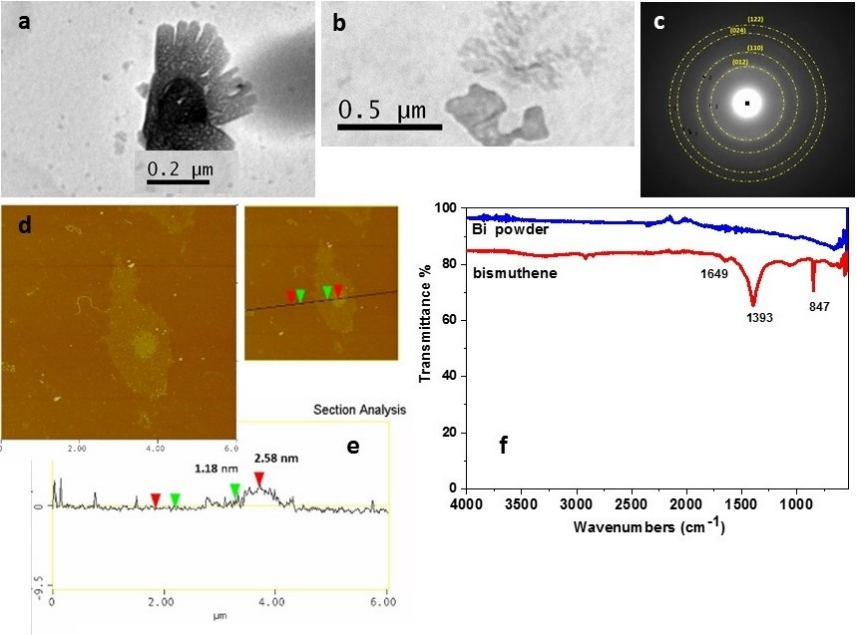
(a,b) TEM images c) SAED pattern, d) AFM image and e) the height profile of bismuthene nanosheets, f) FTIR spectra of bismuthene and Bi powder.

**Bismuthene**. The exfoliation of Bi powder resulted in the production of a milky white colored colloidal dispersion in water, mostly consisting of 2D bismuthene nanosheets, as seen in the TEM and AFM images. The size of the nanosheets ranged between 200 nm and 500 nm (Figure 3a and b). AFM images showed the existence of two‐layer bismuthene nanosheets with a thickness of about 1.2 nm (Figure 3d and e). The thickness of bismuthene monolayer nanosheets has been measured to 0.6 nm.^[48]^ The FTIR spectrum of bismuthene showed three characteristic peaks at 1649, 1393 and 847 cm^−1^, while Bi powder showed no evidence of vibration peaks (Figure 3f). The origin of these vibrations is not clearly defined in the literature, although they have been observed in bismuthene.[^49^, ^50^, ^51^, ^52^, ^53^, ^54^] The first two peaks have been assigned to the carbonyl group[^52^, ^53^] and the third one to Bi−O−Bi vibration on the surface of bismuthene.^[54]^ Alternatively, according to Prodromidis etal the peaks at 1393 and 847 cm^−1^ are attributed to the enhanced absorption of the atmospheric CO_2_ on the enlarged bismuthene surface, indicating the successful exfoliation.^[51]^

**Figure 4:**
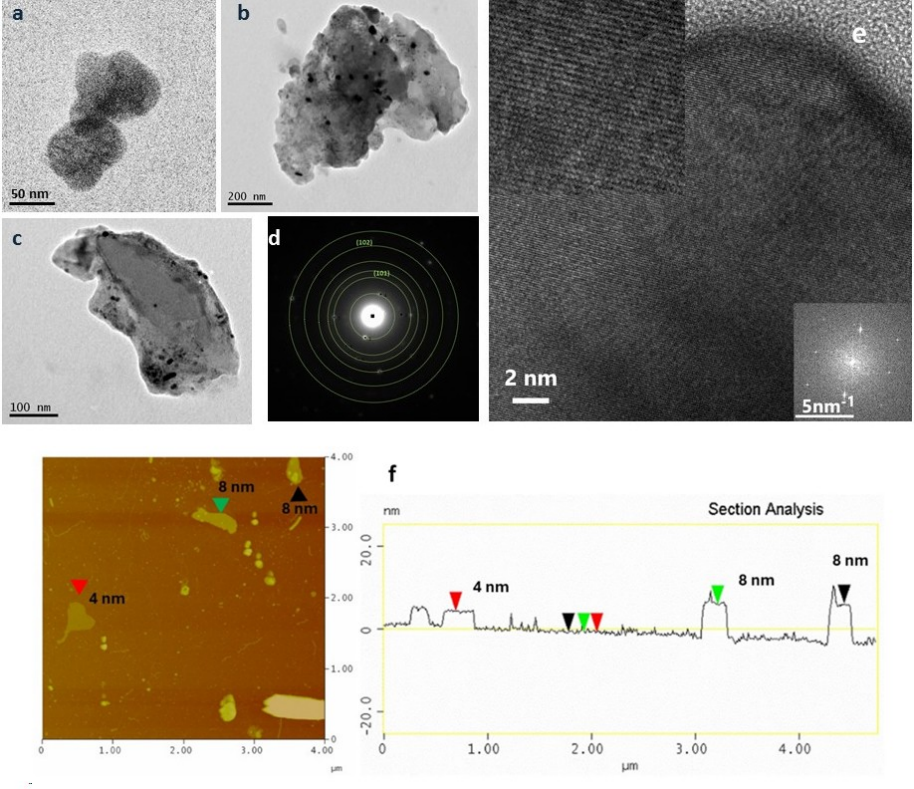
a–c) TEM images, d) SAED pattern, e) HRTEM image of tellurene sample showing TeO_2_ (220) and (004) lattice fringes. Upper left inset: Magnified portion of HRTEM image. Lower right inset: FFT calculated from HRTEM image, f) AFM image of tellurene nanosheets.

**Figure 5:**
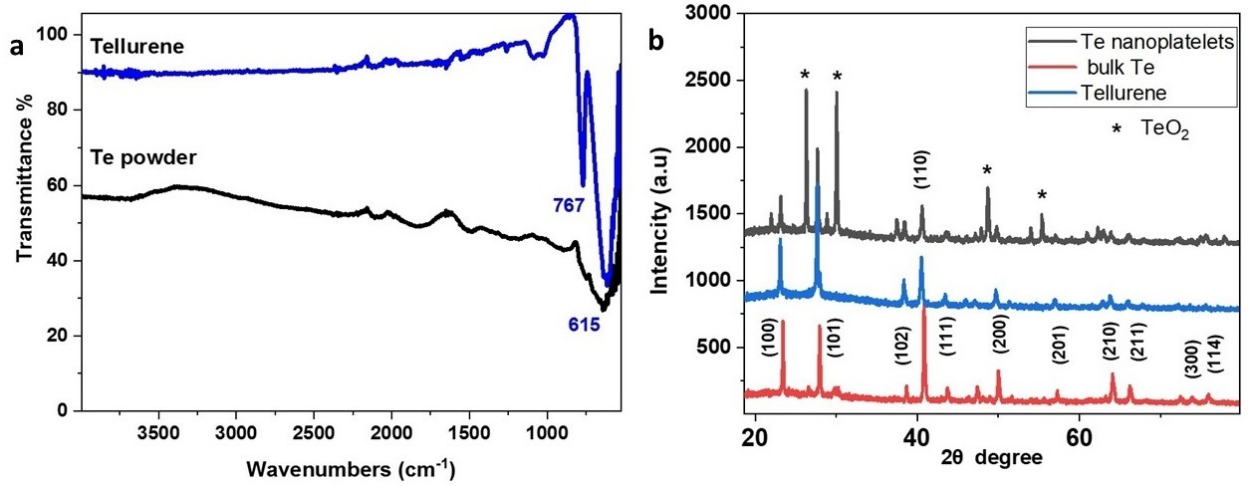
a) FTIR spectra of tellurene and Te powder, b) XRD patterns of bulk Te, oxidized Te nanoplatelets and pure tellurene after reduction.

**Tellurene**. The exfoliation of Te powder resulted in the production of mainly 2D water stable grey colored tellurene nanosheets, as shown by TEM and AFM images. The size of the tellurene nanosheets ranges between 200 nm and a few microns (Figure 4a–c). AFM images showed the existence of thin tellurene nanosheets with a thickness of about 4 nm (Figure 4f and S2). In Figure 4e, a HRTEM image of the tellurene sample can be seen. The (220) and (004) lattice fringes of TeO_2_ (JCPDS no 42–1365) can be seen in the upper left inset showing a magnified part of the HRTEM image. The lattice fringes are normal to each other and their interplanar spacings are 0.170 nm and 0.190 nm respectively as measured by the Fast Fourier Transform calculated from the HRTEM image and shown in the lower right inset. Tellurene showed two intense peaks in the FTIR spectrum at 767 and 615 cm^−1^ which correspond to the symmetrical equatorial and asymmetrical axial frequencies of the Te−O bonds (Figure 5a).^[55]^ The presence of oxygen in the structure of the original Τe powder is also indicated by the presence of the same signals in its spectrum. Τhe peaks are more pronounced in tellurene due to increased oxidation during the exfoliation process.

The XRD pattern and HRTEM images indicated that the gray colored dispersion after the liquid exfoliation of bulk Te consisted of oxidized tellurene nanosheets. Addition of excess sodium borohydride (NaBH_4_) to the aqueous dispersion of oxidized tellurene nanosheets and stirring for few minutes led to their reduction and the formation of a transparent, black‐colored dispersion of pure tellurene as revealed by the XRD pattern (see Figure 5b).

**Figure 6:**
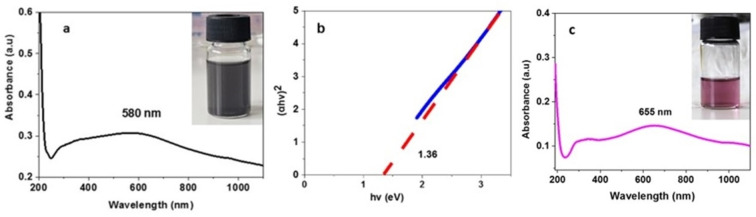
The UV‐Vis spectrum and photo of the aqueous dispersion of a) reduced tellurene nanosheets in aqueous dispersion after treatment with NaBH_4_, b) the Tauc plot for the estimation of the optical bandgap of the reduced tellurene, c) tellurene nanosheets in aqueous dispersion after further treatment of reduced tellurene with NaBH_4_.

After the reduction, the UV‐Vis spectrum of the tellurene nanosheets changed remarkably showing a more intense absorption band with λ_max_=580 nm (see Figure 6a). According to the Tauc plot, the bandgap of the reduced tellurene was estimated to 1.36 eV (see Figure 6b). Further reaction with NaBH_4_ led to a purple‐colored unstable material with λ_max_=655 nm. According to the literature the purple color indicated the formation of Te^4+^ cations.^[56]^

**Figure 7:**
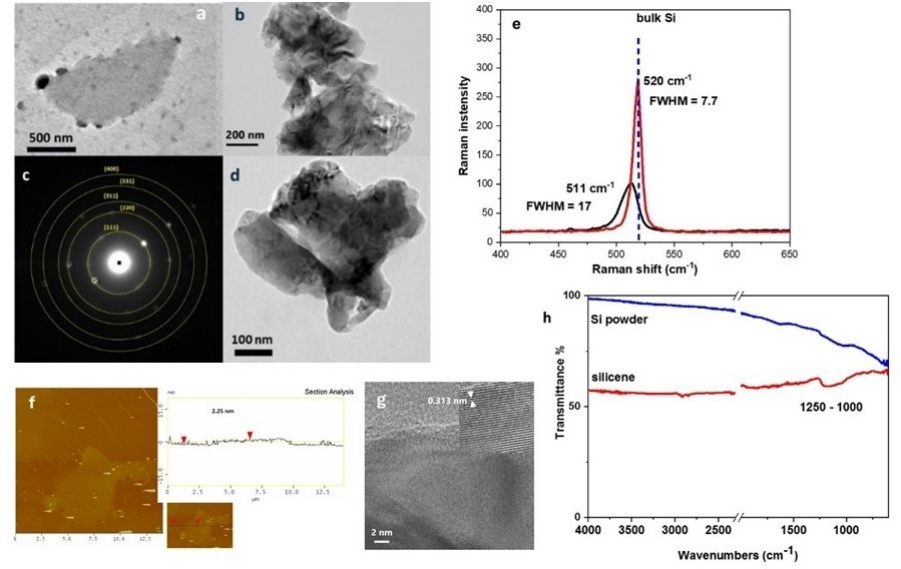
a, b, d) TEM images, c) SAED pattern, e) Raman spectra of silicene nanoplatelets and bulk material, f) AFM image and height profile, g) HRTEM image of silicene nanoplatelets, showing (111) Si lattice fringes. Inset: Magnified portion of HRTEM image exhibiting a lattice spacing of 0.313 nm. h) FTIR spectra of silicene and Si powder.

**Silicene**. After the sonication treatment of bulk Si in aqueous melamine solution, the liquid phase was colored brown due to the formation of silicene nanoplatelets (Figure 1a). As shown in the TEM bright field image of silicene sample in Figure 7a and b, Si forms nanoplatelets of a size about 0.5 to few micrometers. The platelets exhibit diffraction contrast and bend contours. A HRTEM image near the edge of one platelet can be seen in Figure 7g. The (111) lattice fringes of Si with an interplanar spacing of 0.313 nm are clearly observed in the inset. The thickness of silicene nanoplatelets was measured from ΑFM images about 2–4 nm (Figure 7f and S4). The Raman spectrum of bulk Si has a characteristic peak at 520 cm^−1^. The 2D product, silicene nanoplatelets showed a broader peak at 511 cm^−1^ which according to the literature is attributed to nanoscale Si (Figure 7e).[^11^, ^35^] The SAED pattern of silicene indicates the planes (111), (220), (311) (400) and (331) as in the bulk material and the XRD patterns (Figure 7g and S3). The FTIT spectra of silicene and Si powder showed a very low and broad peak between 1000 and 1250 cm^−1^ that corresponds to Si−O−Si vibration of Si surface indicating a very low percentage of oxygen in the silicene product (Figure 7h).

**Figure 8:**
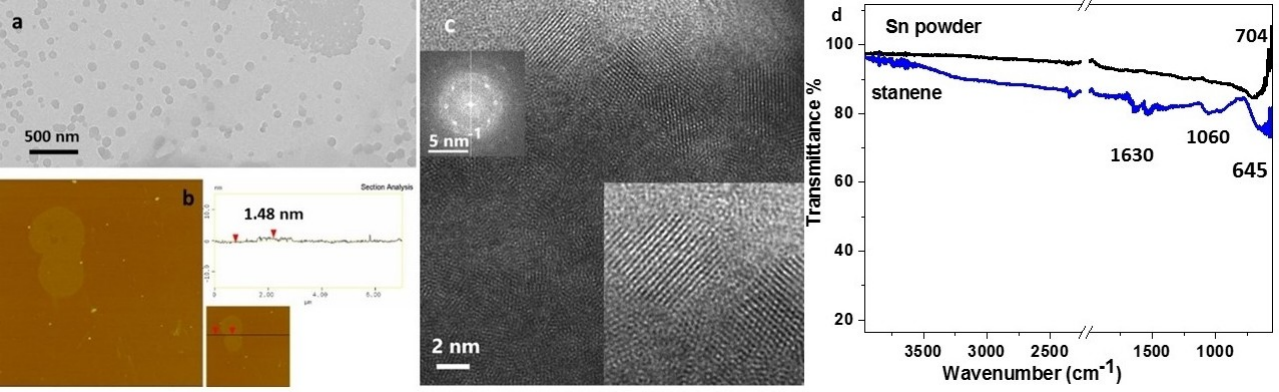
a) TEM, b) AFM image of Sn nanoplatelets and c) HRTEM image of Sn nanoplatelets showing SnO_2_ nanograins. Lower right inset: Magnified portion of HRTEM image exhibiting SnO_2_ (110) lattice fringes. Upper left inset: FFT calculated from HRTEM image. d) FTIR spectra of stanene and Sn powder.

**Stanene**. The exfoliation of Sn powder in water resulted in the formation of a concentrated gray colored colloidal dispersion (see Figure 1d). The TEM images showed mainly small nanoplatelets with a lateral size about 100 nm and a thickness of 1.5 nm according to AFM images (Figure 8b and S6). In Figure 8c, a HRTEM image of the Sn sample is shown. As evidenced by the lower right inset, the particle is composed of nanograins with a size of about 2–5 nm, that are randomly oriented. The interplanar spacing as measured by the Fast Fourier Transform, that was calculated from the HRTEM image and as shown in the lower right inset, is 0.335 nm corresponding to the (110) crystal planes of SnO_2_ (JCPDS 41–1445). The FTIR spectrum of Sn powder showed a peak at 704 cm^−1^ which is attributed to the presence of Sn−O−Sn bonds. Α similar peak shifted to 645 cm^−1^ appears also in stanene slightly increased indicating a limited oxidation of stanene during exfoliation process (Figure 8d). Stanene spectrum showed also peaks at 1060 cm^−1^ (Sn−OH) and around 1630 cm^−1^ (absorbed water molecules)[^57^, ^58^] Liquid exfoliation in water seems to have good yield for all materials ranging between 20 and 50 % and the presence of melamine plays a special role in this. Τhe simplicity of the method which is based on three basic processes such as ultrasound, physical precipitation and centrifugation and on just three components, the starting powder, water and melamine, contribute to the repeatability and reproducibility of the method. The study of the LPE yield and the quality of the final products in different samples showed limited variations confirming the above indications.

A notable advantage of the method described is that the LPE in aqueous melamine solution can be easily applied on a large scale and in a fully sustainable manner. After removal of the prepared nanosheets by centrifugation, the melamine solution can be recycled and used again in the process. Οther green solvents such as Polarclean, and cyrene have similarly high yields in the preparation of 2D materials, mainly TMDs, but have not been widely used for the preparation of Xenes. Αlso, although they have advantages in terms of product quality as they do not favor oxidation, they have the disadvantage of having a high boiling point and are difficult to remove from the final product.[^59^, ^60^, ^61^]

Melamine molecules seem to stabilize the 2D sheets in water and play a positive role in the yield of the exfoliation process, although the mechanism by which melamine interacts with nanosheets has not been fully elucidated. Few examples of the interaction of melamine with 2D materials have been presented in the literature. In a similar process, melamine has been used to exfoliate graphite by ball milling and a small amount of melamine which remain adsorbed promotes the stabilization of graphene in water.^[62]^ The origin of this interaction is traced to the aromatic character of melamine and its planarity that allows *π*,*π* stacking with graphene and the ability of melamine to form 2D networks through hydrogen bonds resembling a 2D polymers.^[63]^

Furthermore, theoretical calculations suggested that the presence of melamine on the surface of graphene contributes to the retention of water molecules very close to the surface of graphene through H‐bonds, thus contributing significantly to its fine dispersion in water.^[64]^ Theoretical studies have shown that organic molecules such as tetrathiafulvalene (TTF) or toluene, benzene or phenol and cresols could be absorbed on 2D nanosheets such as silicene,[^65^, ^66^] stanene^[67]^ or bismuthene^[68]^ respectively while the parallel orientation is stronger and favor the electron transfer between the organic molecules and the nanosheets. The absorbed melamine molecules are probably also responsible for the appearance of energy gap in silicene and similarly in the rest 2D Xenes of this work. The phenomenon of the appearance of an energy gap in silicene by the adsorption of organic molecules has been studied theoretically.[^65^, ^66^]

## 3 Conclusions

In summary, in this work, layered van der Waals solids, silicon and tin as well as non van der Waals, tellurium and bismuth powders were sonicated in aqueous solution of melamine. The result of the procedure was the formation of 2D nanostructures like thin nanoplatelets of silicene and stanene and nanosheets of tellurene and bismuthene. Stanene and tellurene proved to be more sensitive to oxygen, so that their partial oxidation could not be avoided even when the sonication was carried out in deoxygenated water and dried under inert conditions. Resently, it had been shown by our group, that two iron oxides ores, hematite and magnetite, can be effectively exfoliated in an aqueous solution of melamine with the aid of ultrasound, even though they are not layered materials. In this work the ability of melamine in water to assist the exfoliation of materials extends to Xenes and specifically to layered and non‐layered Xenes.

## Experimental Section/Methods

*Materials*. Silicium (99.9 %, Fluka), Tellurium (99–99.5 %, Merck Schuchardt OHG) and Tin (99–99.5 %, Merck Schuchardt OHG) were used as powders. Bismuth pieces, (99.99 % metals basis, Alfa Aesar) were pulverized by griding in mortar. Melamine was purchased from TCI.

*Preparation of 2D nanostructures*. 50 mg of powder was dispersed in a solution of 100 mg of melamine in deoxygenated water. The mixture was sonicated for 2 hours. The mixture was left for one hour to precipitate the unexfoliated metal and the supernatant was filtered (hydrophilic filter membrane nylon 0.2 μm) and washed with water thoroughly. The product was isolated and air dried.

*Electron Microscope Analysis*. The morphology of the products was investigated by transmission electron microscopy (TEM), and high‐resolution TEM (HRTEM). Images were obtained using a JEOL, JEM‐2100 instrument operating at 200 kV and a FEG HR Scanning‐Transmission Electron Microscope (Thermo Fisher Scientific Talos F200iS/TEM). Atomic force microscopy (AFM) images were obtained in tapping mode with a 3D Multimode Nanoscope, using Tap‐300 G silicon cantilevers with a tip radius of <10 nm and a force constant of ≈20–75 N m^−1^. Exfoliated nanosheets or nanoplatelets were deposited onto silicon wafers (P/Bor, single side polished, purchased from Si‐Mat) from aqueous dispersions by drop casting.

*Spectroscopy Measurements*. The UV‐Vis absorption spectra were recorded in water dispersion with a Hitachi Digilab, Model U2800‐Double‐Beam‐UV/Vis. XRD patterns were conducted with a D8 Advance Bruker diffractometer (Bruker AXS, Karlsruhe, Germany) using a CuKa (lD 1.5418) radiation source (40 kV, 40 mA) and a secondary beam graphite monochromator. The laser power was 1.082 mV. Diffraction patterns were recorded in the 2‐theta (20) scale from 2○ to 80○, in steps of 0.02○ and with a counting time of 2 s per step. Raman spectra were recorded with a micro‐Raman spectrometer (HORIBA Jobin‐Yvon T‐64000) using a He−Ne laser source with an excitation wavelength of 632.8 nm. The laser power on the sample was 0.9 mW.

## Conflict of Interests

The authors declare no conflict of interest.

4

## Supporting information

As a service to our authors and readers, this journal provides supporting information supplied by the authors. Such materials are peer reviewed and may be re‐organized for online delivery, but are not copy‐edited or typeset. Technical support issues arising from supporting information (other than missing files) should be addressed to the authors.

Supporting Information

## Data Availability

The data that support the findings of this study are available in the supplementary material of this article.
