# Supplementary material for: Aqueous Dispersion of Xenes by Liquid Phase Exfoliation of Monoelemental Crystals in Melamine Solution
Source: Chemistry. 2024 Dec 11;31(8):e202403770. doi: 10.1002/chem.202403770 (PMC11803363; doi:10.1002/chem.202403770)
Supplement: Supplementary file 1 — Supporting Information [file CHEM-31-e202403770-s001.pdf]

Chemistry–A European Journal

Supporting Information

Aqueous Dispersion of Xenes by Liquid Phase Exfoliation of Monoelemental Crystals in Melamine Solution

Alexia Vaso, Eleni Tegkelidi, Angela S. Kaloudi, Dimitrios P. Gournis, Vasileios Tzitzios, Nikos Boukos, Argiris Kolokithas Ntoukas, and Vasilios I. Georgakilas*

Supplementary Information

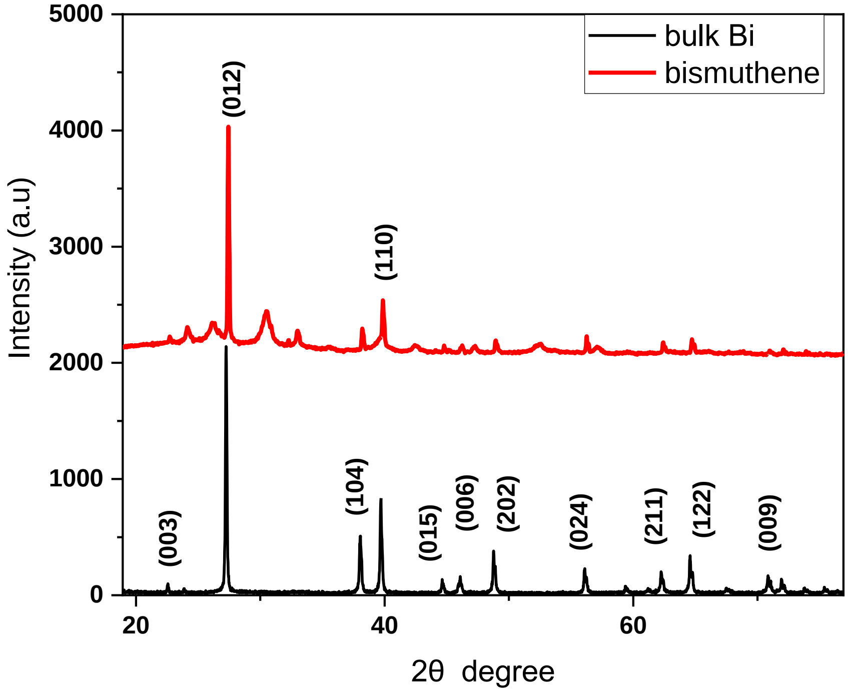

Figure S1. XRD patterns of bulk Bi and bismuthene nanosheets.

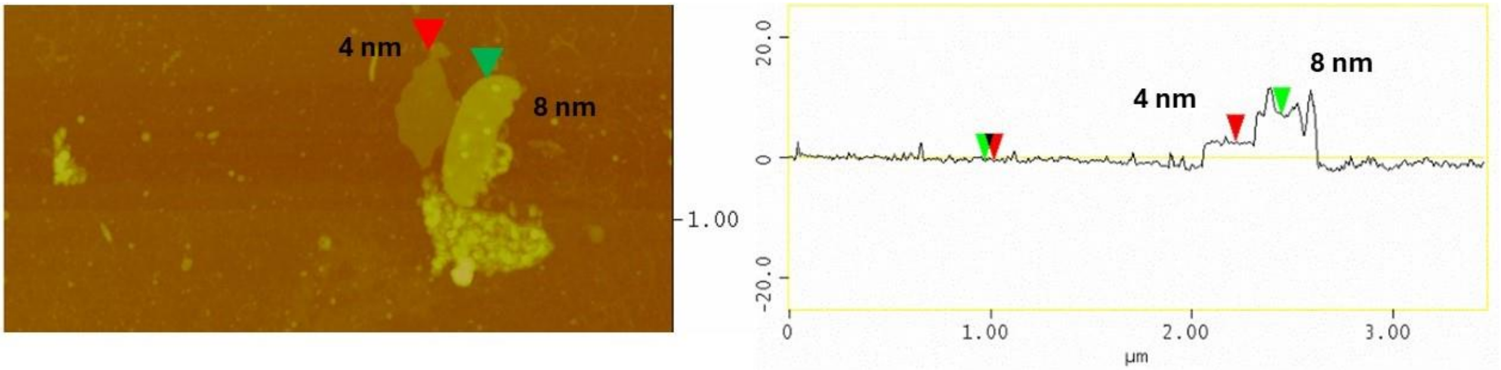

Figure S2. AFM image of tellurene nanosheets.

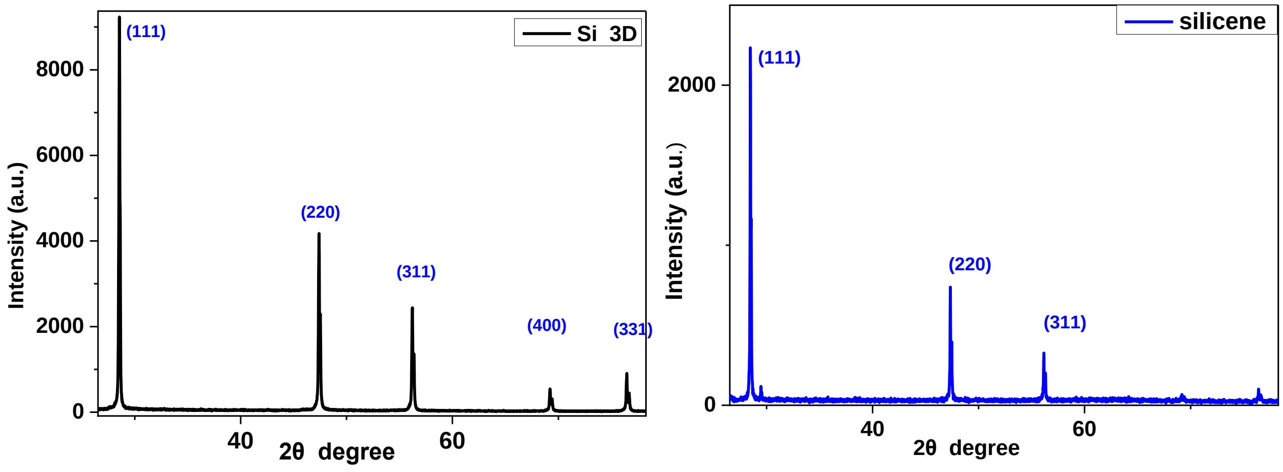

Figure S3. XRD patterns of bulk Si and silicene nanoplatelets.

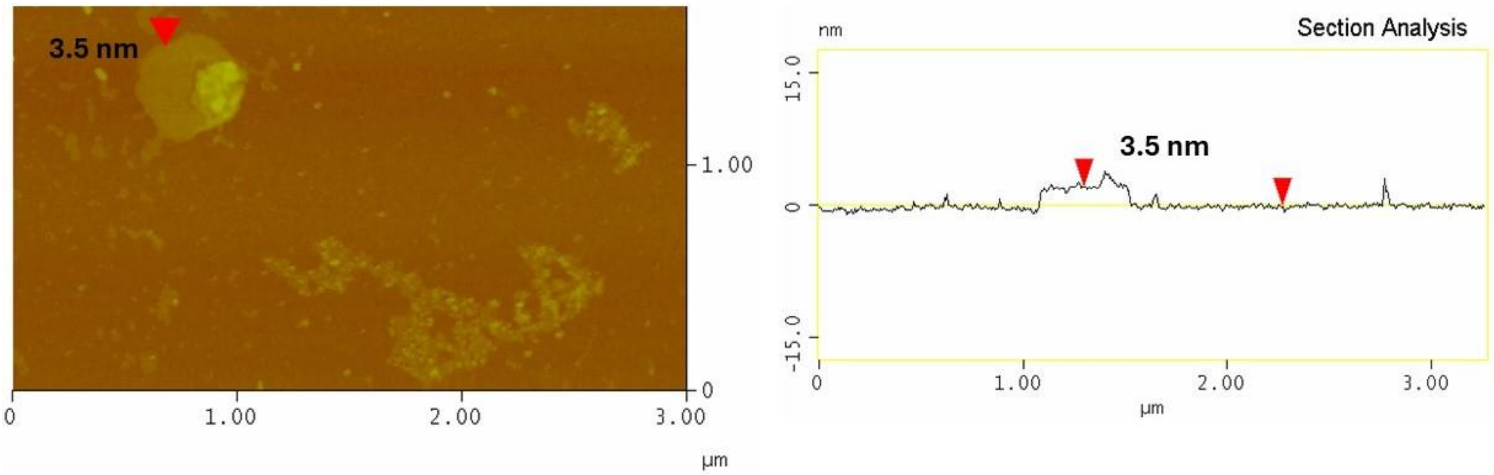

Figure S4. AFM image of silicene nanoplatelets.

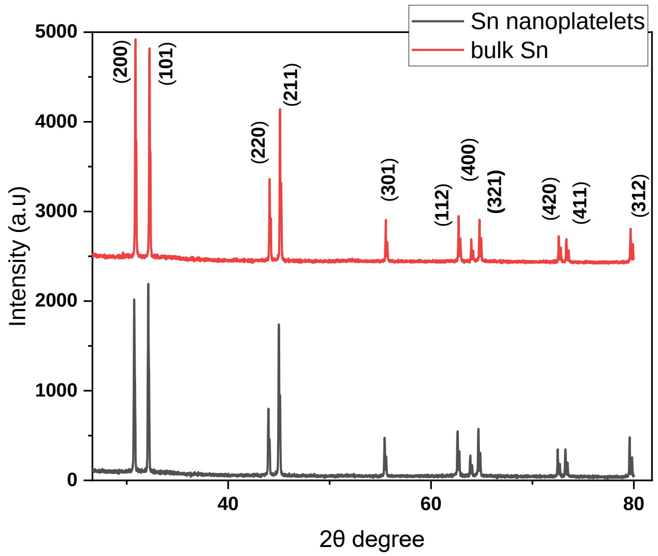

Figure S5. XRD patterns of bulk Sn and stanene nanoplatelets.

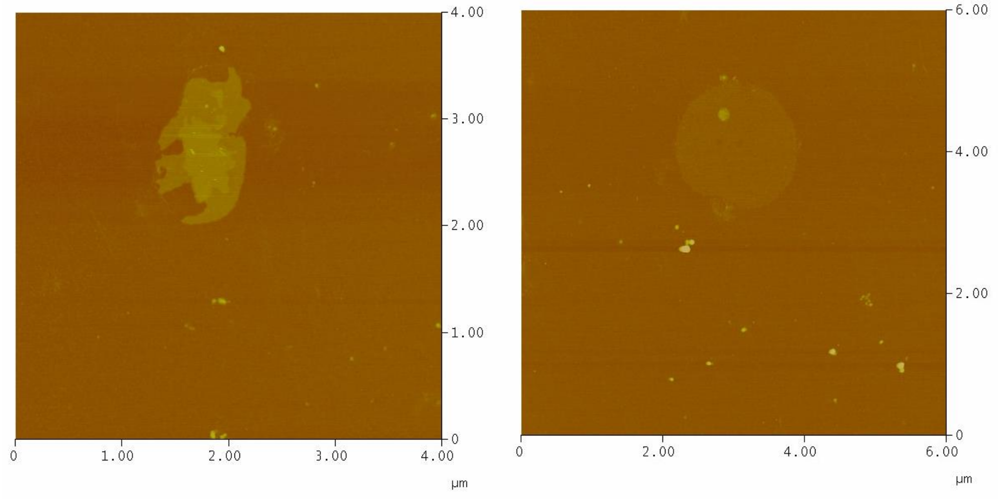

Figure S6. AFM images of stanene nanoplatelets.
